# Cytogenetic Analysis of the Asian Box Turtles of the Genus *Cuora* (Testudines, Geoemydidae)

**DOI:** 10.3390/genes12020156

**Published:** 2021-01-25

**Authors:** Lorenzo Clemente, Sofia Mazzoleni, Eleonora Pensabene, Tomáš Protiva, Philipp Wagner, Uwe Fritz, Lukáš Kratochvíl, Michail Rovatsos

**Affiliations:** 1 Department of Ecology, Faculty of Science, Charles University, 12844 Prague, Czech Republic; lorenzo.clemente@natur.cuni.cz (L.C.); sofia.mazzoleni@natur.cuni.cz (S.M.); pensabee@natur.cuni.cz (E.P.); lukas.kratochvil@natur.cuni.cz (L.K.); 2 Independent Researcher, 14200 Prague, Czech Republic; info@landsnails.org; 3 Allwetterzoo Münster, 48161 Münster, Germany; wagner@allwetterzoo.de; 4 Museum of Zoology, Senckenberg, 01109 Dresden, Germany; uwe.fritz@senckenberg.de

**Keywords:** C-banding, *Cuora*, evolution, FISH, Geoemydidae, heterochromatin, karyotype, microsatellites, rDNA, telomeres

## Abstract

The Asian box turtle genus *Cuora* currently comprises 13 species with a wide distribution in Southeast Asia, including China and the islands of Indonesia and Philippines. The populations of these species are rapidly declining due to human pressure, including pollution, habitat loss, and harvesting for food consumption. Notably, the IUCN Red List identifies almost all species of the genus *Cuora* as Endangered (EN) or Critically Endangered (CR). In this study, we explore the karyotypes of 10 *Cuora* species with conventional (Giemsa staining, C-banding, karyogram reconstruction) and molecular cytogenetic methods (*in situ* hybridization with probes for rDNA loci and telomeric repeats). Our study reveals a diploid chromosome number of 2n = 52 chromosomes in all studied species, with karyotypes of similar chromosomal morphology. In all examined species, rDNA loci are detected at a single medium-sized chromosome pair and the telomeric repeats are restricted to the expected terminal position across all chromosomes. In contrast to a previous report, sex chromosomes are neither detected in *Cuora* *galbinifrons* nor in any other species. Therefore, we assume that these turtles have either environmental sex determination or genotypic sex determination with poorly differentiated sex chromosomes. The conservation of genome organization could explain the numerous observed cases of interspecific hybridization both within the genus *Cuora* and across geoemydid turtles.

## 1. Introduction

Karyotypes have been described in only about 50% [1,2,3] of 363 recognized species of turtles [4,5,6,7,8]. Generally, turtles have rather conserved karyotypes, even though chromosome numbers range from 2n = 26 to 2n = 68 [1,9,10]. Phylogenetic studies suggested that this variability evolved from the putative ancestral karyotype with 2n = 52 chromosomes due to chromosomal rearrangements largely involving microchromosomes [10,11,12]. Nevertheless, the karyotype with 2n = 52 chromosomes remains the most common in cryptodiran turtles [1,13].

Turtles are remarkable for different sex determination modes. Two different systems of sex determination are recognized in amniotes: environmental sex determination (ESD) and genotypic sex determination (GSD) [14,15]. ESD is characterized by the absence of consistent differences in genotypes between sexes; the sex of an individual is determined by environmental factors during a sensitive period of embryonic development [14,15]. ESD is the most common sex determination mechanism in turtles [1,16,17] and is considered ancestral for turtles [13,14,16,17] and possibly also for amniotes as a whole [14,15]. Under GSD, the sex of an individual is determined by its sex-specific genotype, i.e., by a combination of sex chromosomes at conception. In turtles, GSD likely evolved at least five times [16,17,18]. Female heterogamety (ZZ/ZW sex chromosomes) is known only in softshell turtles (Trionychidae), while male heterogamety (XX/XY sex chromosomes) occurs in turtles of the family Chelidae and in the genera *Staurotypus* (Kinosternidae) and *Glyptemys* (Emydidae) as well as in *Siebenrockiella crassicollis* (Geoemydidae) [1,19,20,21,22,23,24,25,26,27]. Heteromorphic ZZ/ZW sex chromosomes were reported also in *Pangshura smithii* [28]. However, a recent investigation revealed that the sex chromosome identification was erroneously based on chromosome pairing during karyotype reconstruction [17].

With 71 described species, the family Geoemydidae is the most diverse turtle family [8,29,30]. Despite their wide geographical range, the cytogenetic characteristics of this family are still poorly studied. Karyotypes have been described only in 41 species [1,2,3,12,22,31,32,33,34,35,36]. The chromosome numbers vary between 2n = 50 to 2n = 56, with 2n = 52 being the most common [17,22,32,34,36,37]. C-banding performed in *Geoemyda spengleri*, *Geoemyda japonica*, *Pangshura smithii*, *Rhinoclemmys punctularia* and *Siebenrockiella crassicollis* revealed accumulation of heterochromatin in microchromosomes, and in centromeric and pericentromeric regions of macrochromosomes [17,22,36]. Both ESD and GSD systems have been reported in the family Geoemydidae: evidence for ESD was provided for species from the genera *Mauremys*, *Heosemys*, *Rhinoclemmys,* and *Cuora* [38,39], while XX/XY system with heteromorphic sex chromosomes is known in *Siebenrockiella crassicollis* [22].

The Asian box turtles of the genus *Cuora* are arranged into 13 species distributed in Southeast Asia, including China, Indonesia, and Philippines [8,29,30]. All *Cuora* species are rapidly declining due to human pressure, including pollution, habitat loss, and unsustainable harvesting for food consumption [4,29,40]. All species are threatened with extinction and are classified as Endangered (EN) or Critically Endangered (CR) by the IUCN [41]. To our knowledge, only six out of 13 species have been examined cytogenetically. The karyotype of 2n = 50 has been described for *Cuora amboinensis* in early pioneering studies [32,42], but it was later revised to 2n = 52 [33]. A diploid chromosome number of 2n = 52 chromosomes was also reported in *Cuora aurocapitata* [34], *Cuora flavomarginata* [12,32], *Cuora galbinifrons* [34], *Cuora mouhotii* (originally reported under its previous genus name *Pyxidea mouhotii*) [32], and *Cuora trifasciata* [33]. ESD was found in *Cuora flavomarginata* [39] and heteromorphic XX/XY sex chromosomes, corresponding to the sixth largest chromosome pair, have been reported in *Cuora galbinifrons* by conventional cytogenetic methods, but figures of karyograms or metaphases were not provided [34]. Sex chromosomes were not found in any other examined species of the genus *Cuora* [12,32,33,34], and the presence of sex chromosomes in *Cuora galbinifrons* should thus be reexamined.

In this study, we examined 10 species from the genus *Cuora*, intending to provide a comprehensive cytogenetic analysis of this threatened group of turtles. We reconstructed karyograms and explored the distribution of the constitutive heterochromatin, the rDNA loci, and the TTAGGG telomeric repeats. In addition, we focused on exploring the presence of sex chromosomes.

## 2. Materials and Methods

### 2.1. Material

Blood samples were collected from 10 species of Asian box turtles (Table 1), either from the dorsal coccygeal vein or the subcarapacial vein. The blood samples were used for DNA extraction and preparation of mitotic chromosome suspensions. All turtles used in this study were kept in Allwetterzoo Münster (Germany), the Museum of Zoology, Senckenberg Dresden (Germany), and private breeders.

### 2.2. Taxonomic Verification

The species identity of all studied turtles was determined using external morphology. In addition, we sequenced for each studied species the mitochondrial gene *cytochrome b* (*cytb*). Total DNA was extracted with the DNeasy Blood and Tissue Kit (Qiagen, Valencia, CA, USA). The *cytb* gene was amplified by PCR using primers designed by Burbrink et al. [43]. The PCR products were purified and sequenced bi-directionally by Macrogen (Seoul, South Korea). The sequences were trimmed in FinchTV [44], analyzed in Geneious Prime [45] and compared to sequences deposited in public databases by BLASTn [46] to corroborate morphological determinations. All sequences were deposited in GenBank.

### 2.3. Chromosomes Preparation, Staining, and C-Banding

Chromosomal suspensions were prepared following the protocol described in Mazzoleni et al. [15]. Briefly, the blood was cultured for a week at 30 °C in the medium composed of 10% fetal bovine serum (Gibco, Thermo Fisher Scientific Inc., Waltham, MA, USA), 0.5% penicillin/streptomycin solution (Gibco, Thermo Fisher Scientific Inc., Waltham, MA, USA), 1% L-glutamine solution (Sigma-Aldrich, St. Louis, MO, USA), 3% phytohemagglutinin (Gibco, Thermo Fisher Scientific Inc., Waltham, MA, USA), and 1% lipopolysaccharide solution (Sigma-Aldrich, St. Louis, MO, USA). Chromosome preparations were made following standard procedure including 3.5 h treatment with colchicine, 30 min treatment with 0.075 M KCl solution, and four rounds of fixation with cold 3:1 methanol: acetic acid solution.

Chromosome suspensions were spread to slides, and were incubated at 55 °C for 1 h, prior to all cytogenetic stains. The distribution of constitutive heterochromatin was detected by C-banding, with a protocol previously described in Mazzoleni et al. [15], based on the protocol of Sumner [47].

### 2.4. Giemsa Staining and Karyogram Construction

The slides were stained with 5% Giemsa solution. Selected metaphases were captured using a Zeiss Axio Imager Z2 (Zeiss, Oberkochen, Germany), equipped with a Metafer-MSearch automatic scanning platform (MetaSystems, Altlussheim, Germany) and CoolCube 1 b/w digital camera (MetaSystems, Altlussheim, Germany). At least 20 metaphases per individual were analyzed. Karyograms were constructed using Ikaros karyotyping software (MetaSystems, Altlussheim, Germany).

### 2.5. Fluorescence In Situ Hybridization (FISH)

The probe for rDNA loci was prepared from a plasmid encoding the 18S and 28S rRNA units of *Drosophila melanogaster* [48] and labeled with biotin-dUTP using a Nick Translation Kit (Abbott Laboratories, Chicago, IL, USA). The probe with the telomeric motif (TTAGGG)_n_ was prepared and labeled with dUTP-biotin by PCR, using the primers (TTAGGG)_5_ and (CCCTAA)_5_, without a DNA template [49,50].

Fluorescence in situ hybridization was performed following the procedure detailed in Mazzoleni et al. [15]. The chromosomal preparations were treated with RNase A and pepsin, fixed with 1% formaldehyde, dehydrated through a series of 70%, 85%, and 100% ethanol washes, denatured in 70% formamide/2 × SSC at 75 °C for 3 min and dehydrated again. Hybridization with 11 µL of probe was performed at 37 °C overnight. Post-hybridization washes were performed in 50% formamide/2 × SSC at 42 °C and in 2 × SSC. Slides were incubated in 100 μL of 4 × SSC/5% blocking reagent (Roche, Basel, Switzerland) at 37 °C for 45 min and then in 4 × SSC/5% blocking reagent containing avidin-FITC (Vector laboratories, Burlingame, CA, USA) for 30 min at 37 °C. The fluorescence signal was twice amplified by the fluorescein–avidin D/biotinylated anti-avidin system (Vector Laboratories, Burlingame, CA, USA). After this treatment, the slides were dehydrated in ethanol series, air-dried and stained with Fluoroshield with DAPI (Sigma-Aldrich, St. Louis, MO, USA).

For each specimen, at least 20 images were obtained using a Provis AX70 (Olympus, Tokyo, Japan) fluorescence microscope equipped with a DP30BW digital camera (Olympus, Tokyo, Japan). The photos were superimposed in color and further processed with DP Manager imaging software (Olympus, Tokyo, Japan).

## 3. Results

**Figure 1:**
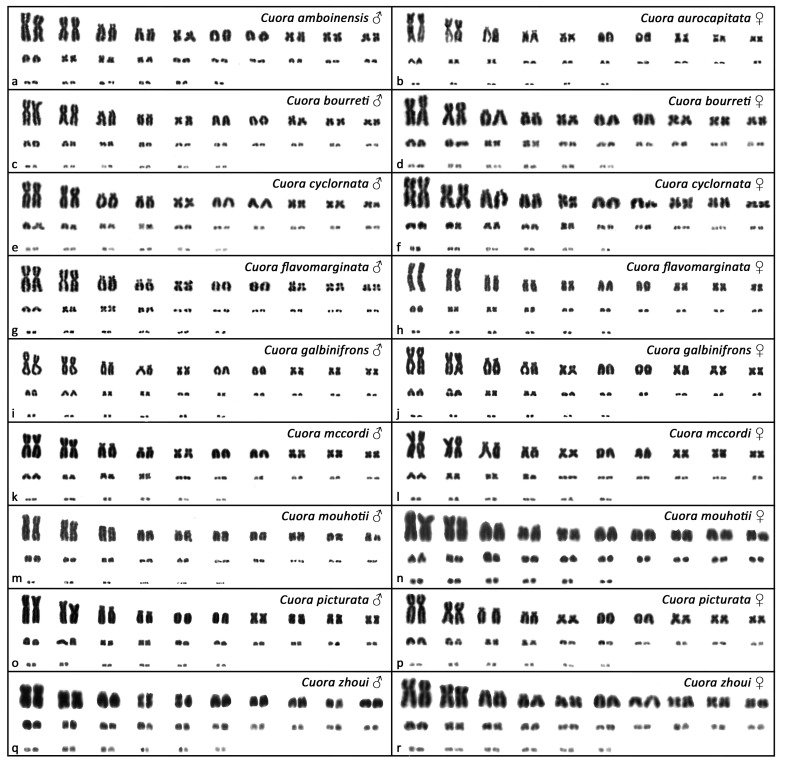
Karyograms from Giemsa-stained metaphases of Cuora amboinensis (**a**), Cuora aurocapitata (**b**), Cuora bourreti (**c**,**d**), Cuora cyclornata (**e**,**f**), Cuora flavomarginata (**g**,**h**), Cuora galbinifrons (**i**,**j**), Cuora mccordi (**k**,**l**), Cuora mouhotii (**m**,**n**), Cuora picturata (**o**,**p**), and Cuora zhoui (**q**,**r**).

**Figure 2:**
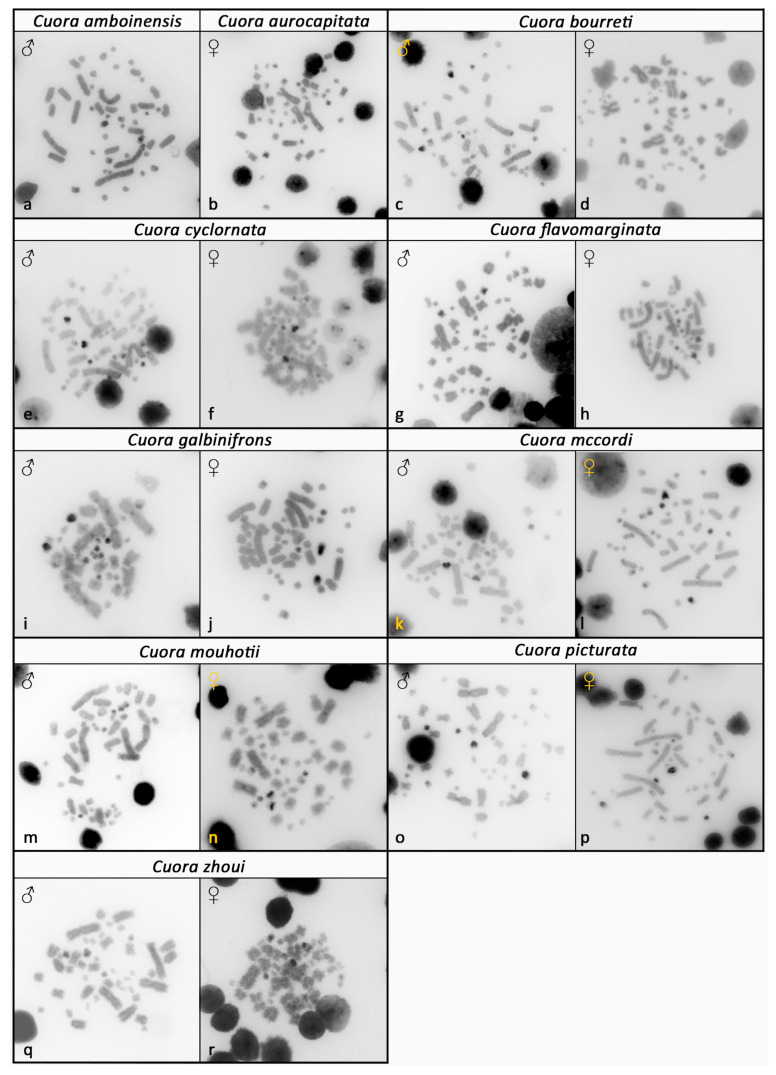
C-banded metaphases of Cuora amboinensis (**a**), Cuora aurocapitata (**b**), Cuora bourreti (**c**,**d**), Cuora cyclornata (**e**,**f**), Cuora flavomarginata (**g**,**h**), Cuora galbinifrons (**i**,**j**), Cuora mccordi (**k**,**l**), Cuora mouhotii (**m**,**n**), Cuora picturata (**o**,**p**), and Cuora zhoui (**q**,**r**). Prominent heterochromatic blocks were detected in both chromosomes of the 14th pair in all species as well as in other smaller microchromosomes.

**Figure 3:**
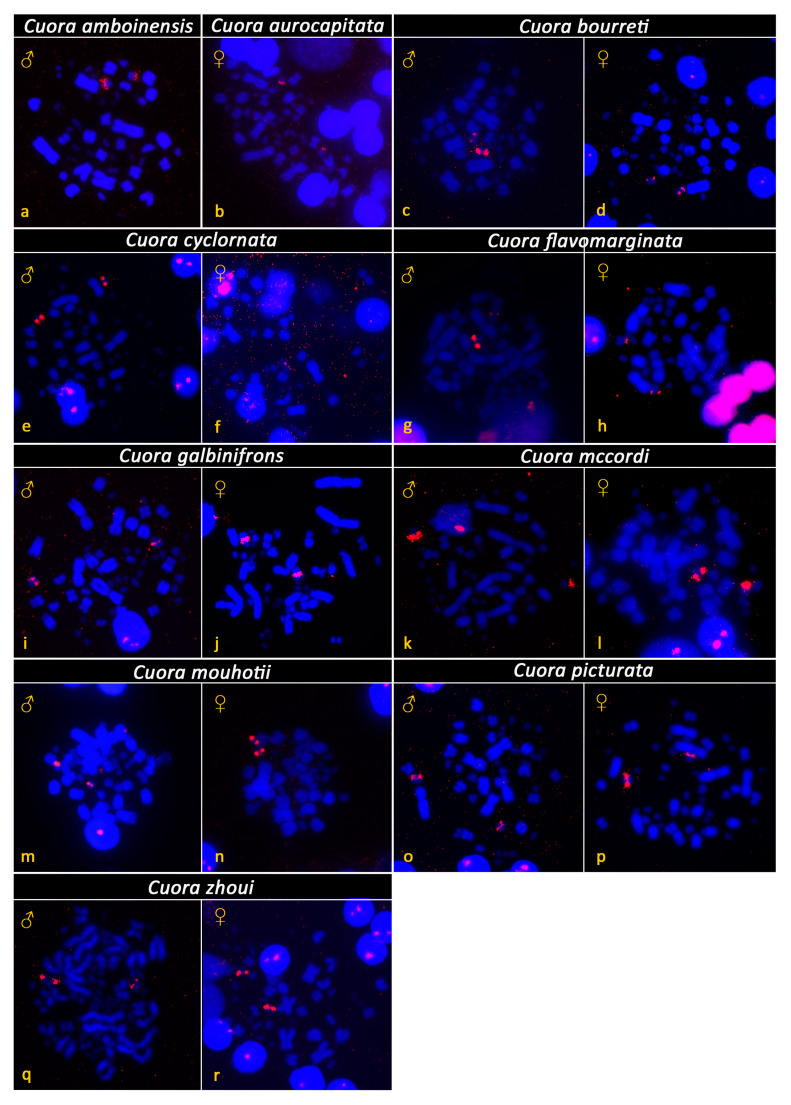
Distribution of rDNA loci (red color) on DAPI-stained metaphases (blue color) from *Cuora amboinensis* (**a**), *Cuora aurocapitata* (**b**), *Cuora bourreti* (**c**,**d**), *Cuora cyclornata* (**e**,**f**), *Cuora flavomarginata* (**g**,**h**), *Cuora galbinifrons* (**i**,**j**), *Cuora mccordi* (**k**,**l**), *Cuora mouhotii* (**m**,**n**), *Cuora picturata* (**o**,**p**), and *Cuora zhoui* (**q**,**r**). rDNA loci accumulate in the 9th chromosomal pair, in all examined species.

**Figure 4:**
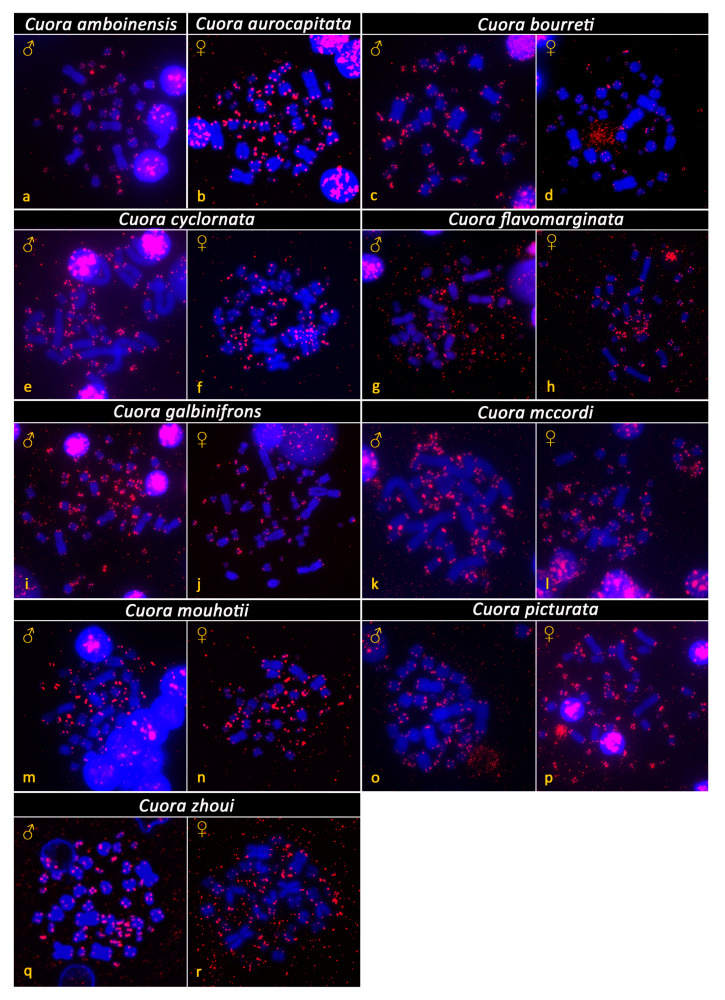
Distribution of telomeric repeats (red color) on DAPI-stained metaphases (blue color) from *Cuora amboinensis* (**a**), *Cuora aurocapitata* (**b**), *Cuora bourreti* (**c**,**d**), *Cuora cyclornata* (**e**,**f**), *Cuora flavomarginata* (**g**,**h**), *Cuora galbinifrons* (**i**,**j**), *Cuora mccordi* (**k**,**l**), *Cuora mouhotii* (**m**,**n**), *Cuora picturata* (**o**,**p**), and *Cuora zhoui* (**q**,**r**). Telomeric repeats have the expected topology at the edges of the chromosomes in all examined species. Interstitial telomeric repeats were not detected.

**Figure 5:**
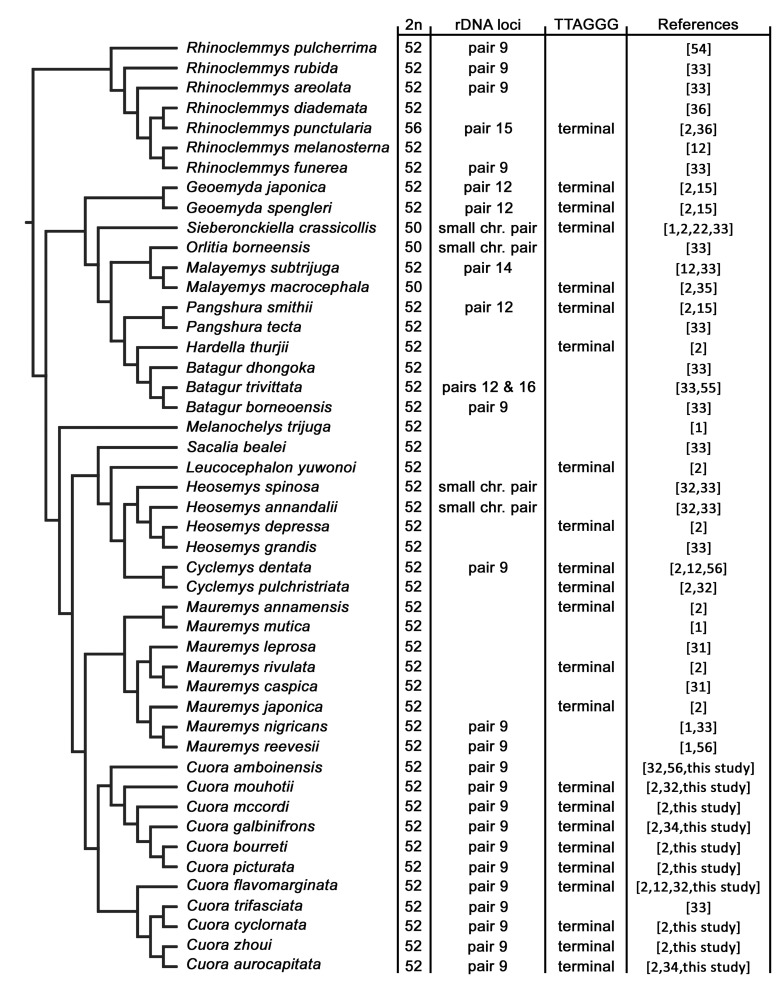
Distribution of diploid chromosome numbers and topology of rDNA loci and TTAGGG telomeric repeats across the phylogeny of geoemydid species. Phylogenetic branching patterns from Colston et al. [30].

All examined species of Asian box turtles showed karyotypes with 2n = 52 and a similar chromosome morphology between homologous pairs (Figure 1). Their karyotypes consisted of both macrochromosomes (pairs 1 to 10) and microchromosomes (pairs 11 to 26), with a gradual decrease in size. Most macrochromosomes were bi-armed, with the exception of the pairs 6 and 7, which were acrocentric. The morphology of microchromosomes was hard to distinguish. Two relatively bigger microchromosomes, assigned as the 14th pair, were fully heterochromatic in all tested species, as revealed by C-banding (Figure 2). Additional heterochromatic blocks were detected in up to three pairs of microchromosomes (Figure 2). A strong accumulation of rDNA loci was detected in both chromosomes of the 9th pair in all tested species (Figure 3). Telomeric motifs were detected in the expected terminal position of all chromosomes (Figure 4 and Figure 5). Notably, sex-specific differences were not detected in the eight species where both sexes were examined (Figure 1, Figure 2, Figure 3 and Figure 4). In addition, a heteromorphic pair of chromosomes was not detected in *Cuora amboinensis* and *Cuora aurocapitata*, where only a single sex was examined (Figure 1, Figure 2, Figure 3 and Figure 4).

## 4. Discussion and Conclusions

The different species of the genus *Cuora* show remarkably similar karyotypes with 2n = 52 chromosomes and a similar topology of rDNA loci and telomeric repeats. Our karyotype reconstructions confirmed the chromosome number and morphology reported in the previous studies for *Cuora amboinensis*, *Cuora aurocapitata*, *Cuora flavomarginata*, *Cuora galbinifrons*, and *Cuora mouhotii* [12,32,33,34] (Figure 1). To our knowledge, the karyotypes of *Cuora bourreti*, *Cuora cyclornata*, *Cuora mccordi*, *Cuora picturata*, and *Cuora zhoui* are described here for the first time.

In a wider phylogenetic context, the diploid chromosome number of 2n = 52 seems to be ancestral not only for the genus *Cuora*, but also for the clade Testudinoidea, consisting of the families Geoemydidae (Figure 5), Emydidae, Platysternidae, and Testudinidae, where it is also the most common chromosome number [1,2]. In comparison to other amniotes, turtles show a slow rate of chromosomal rearrangements and karyotype evolution [1,13], which was also confirmed by comparative examination of genome assemblies at the chromosome level in the turtles *Gopherus evgoodei*, *Chrysemys picta*, and *Trachemys scripta* [51,52,53].

The rDNA loci are commonly detected in a single pair of small chromosomes in both cryptodiran and pleurodiran turtles, i.e., in the two deeply divergent suborders of extant turtles (Figure 5) [1,17,26,32,33,35,36,54,55,56,57]. The same topology was revealed for the genus *Cuora* (Figure 3 and Figure 5). In fact, rDNA loci were detected in the 9th chromosome pair in previous studies for *Cuora amboinensis* and *Cuora trifasciata* [33], as well as in several other geoemydids from the genera *Batagur*, *Cyclemys*, *Mauremys*, and *Rhinoclemmys* [12,33,56] (Figure 5).

Telomeric repeats were detected only in the terminal positions of the chromosomes in all species that we examined. In contrast to other amniotes, such as squamate reptiles, birds, and mammals [2,50,58,59], turtles rarely show interstitial telomeric repeats (ITRs). In fact, ITRs were detected only in 10 out of 65 species of turtles [2], supporting that chromosomal rearrangements are probably less frequent in turtles in comparison to other lineages of amniotes. Nevertheless, we should keep in mind that the correlation between rates of chromosomal rearrangements and ITRs is not absolute. In some lineages, for example, in falcons and eagles, extensive chromosomal rearrangements were not connected with an emergence of notable accumulations of interstitial telomeric sequences [58,60].

The similarity in chromosome number and genome organization in *Cuora* species, and in turtles in general, can partially explain the numerous cases of interspecific hybridization in this genus. Notably, successful hybridization was previously documented among several geoemydid species, involving even phylogenetically highly distant genera (e.g., *Cuora* x *Mauremys*, *Cuora* x *Sacalia*, *Cyclemys* x *Mauremys*, *Mauremys* x *Sacalia*) [61,62,63,64,65,66,67].

In a previous study, XX/XY sex determination was reported for *Cuora galbinifrons* [34], but we did not detect sex chromosomes, either for this or for any other species of Asian box turtles, despite using the same conventional cytogenetic methods as Guo et al. [34] and, additionally, molecular cytogenetic approaches. Taking into consideration that Guo et al. [34] did not provide further evidence such as karyograms or illustrations of metaphases, we can only speculate that autosomes were probably misidentified as sex chromosomes.

We demonstrated that the Asian box turtles of the genus *Cuora* share many cytogenetic characteristics: karyotypes with 2n = 52 chromosomes, similar chromosome morphology, distribution of heterochromatin, rDNA loci, and telomeric repeats. The combination of conventional and molecular cytogenetic analyses did not reveal differentiated sex chromosomes, in contrast to a previous study [34], where sex chromosomes were reported for *Cuora galbinifrons* by applying similar methods. We assume that turtles of the genus *Cuora* have either ESD, as documented for *Cuora flavomarginata* [39], or GSD with poorly differentiated sex chromosomes. To corroborate this, we propose further studies. In particular, the variation of hatchling sex ratio should be examined under laboratory conditions, including the controlled incubation of eggs under a wide range of temperatures and the study of the hatchlings’ sex ratio. If the results of such experiments suggest the presence of GSD, the application of modern next generation sequencing methodologies (DNAseq, RADseq) should be used to identify the cytogenetically undistinguishable sex chromosomes.

## Figures and Tables

**Table 1:** Number of Asian box turtles per species and sex, analyzed in the current study.

Species	♂	♀
*Cuora amboinensis*	1	-
*Cuora aurocapitata*	-	3
*Cuora bourreti*	1	1
*Cuora cyclornata*	1	1
*Cuora flavomarginata*	1	1
*Cuora galbinifrons*	2	2
*Cuora mccordi*	1	1
*Cuora mouhotii*	2	3
*Cuora picturata*	1	1
*Cuora zhoui*	2	3

## Data Availability

All relevant data are provided in the manuscript.
